# Fragilidade entre Pacientes não Idosos Submetidos à Cirurgia Cardíaca

**DOI:** 10.36660/abc.20190082

**Published:** 2020-10-13

**Authors:** Camila Bottura, Livia Arcêncio, Hannah Miranda Araújo Chagas, Paulo Roberto Barbosa Evora, Alfredo José Rodrigues

**Affiliations:** 1 Universidade de São Paulo Faculdade de Medicina de Ribeirão Preto São PauloSP Brasil Faculdade de Medicina de Ribeirão Preto - Universidade de São Paulo, Ribeirão Preto, São Paulo, SP - Brasil

**Keywords:** Fragilidade, Revascularização Miocárdica/cirurgia, Valvas Cardíacas/cirurgia, Cuidados Pós-Operatórios/mortalidade

## Abstract

**Fundamento:**

Geralmente vista como uma característica da velhice, a fragilidade também pode ocorrer em pessoas não idosas, principalmente naquelas que sofrem de doenças crônicas. A fragilidade pode aumentar o risco operatório.

**Objetivos:**

Determinar a prevalência de fragilidade em pacientes submetidos à cirurgia de revascularização do miocárdio (CRM) e/ou troca ou reconstrução valvar e/ou cirurgia valvar, bem como a influência da fragilidade nos desfechos pós-operatórios.

**Métodos:**

Nosso estudo incluiu 100 adultos que foram submetidos a operações cardíacas eletivas consecutivas. A fragilidade foi avaliada por meio da escala de Fried. Os pacientes também realizaram um teste de caminhada de 6 minutos, e medimos as pressões inspiratória e expiratória máximas. Um valor de p < 0,05 foi considerado significativo.

**Resultados:**

De uma coorte de 100 pacientes, com base nos critérios de fragilidade de Fried, 17 pacientes (17%) foram considerados frágeis, 70 (70%) pré-frágeis e apenas 13 (13%) não frágeis. Entre os portadores de valvopatia, 11 (18,6%) foram considerados frágeis e 43 (73%) pré-frágeis. Cinquenta e três por cento dos pacientes considerados frágeis tinham menos de 60 anos (mediana=48 anos). As diferenças no fenótipo de fragilidade entre os pacientes com valvopatia e doença arterial coronariana não foram estatisticamente significativas (p=0,305). A comparação entre pacientes não frágeis, pré-frágeis e frágeis não mostrou diferença significativa na distribuição das comorbidades e do estado funcional cardíaco, independentemente da doença cardíaca. No entanto, a mortalidade hospitalar mostrou-se significativamente maior em pacientes frágeis (29,4%, p=0,026) que em pacientes pré-frágeis (8,6%) e não frágeis (0%).

**Conclusões:**

A fragilidade é prevalente mesmo entre pacientes não idosos submetidos a CRM ou cirurgia cardíaca valvar e está associada a maior mortalidade hospitalar pós-operatória.

## Introdução

A fragilidade é uma síndrome de maior vulnerabilidade a fatores de estresse, incluindo hospitalização, e está associada a uma redução da reserva fisiológica secundária ao declínio no funcionamento ótimo de múltiplos sistemas fisiológicos, o que predispõe os indivíduos a alto risco de eventos adversos.^1^ É uma síndrome multidimensional que compreende dimensões físicas, psicológicas e sociais,^2^ geralmente vista principalmente como uma síndrome geriátrica^3^ caracterizada por baixa atividade física, fraqueza muscular, desempenho lento, fadiga ou baixa resistência e perda de peso não intencional. Neste contexto, os critérios de Fried são amplamente utilizados para os domínios físicos da fragilidade, que podem ser facilmente interpretados por não geriatras e podem ter valor prognóstico.^4^^,^^5^ Demonstrou-se associação de fragilidade com comorbidades crônicas.^2^^,^^6^ Portanto, até mesmo pacientes não idosos podem apresentar essa condição.

Observamos, ao longo de nossa prática clínica, que vários pacientes não idosos submetidos à cirurgia cardíaca a céu aberto apresentam quadro clínico compatível com fragilidade, geralmente apresentando desfechos hospitalares pós-operatórios menos favoráveis.

Portanto, o objetivo do presente estudo foi determinar a prevalência de fragilidade em pacientes não idosos submetidos à revascularização do miocárdio (CRM) e/ou cirurgia valvar, bem como avaliar a influência da fragilidade nos resultados hospitalares pós-operatórios.

### Pacientes e Métodos

#### Desenho do Estudo e Participantes

Estudamos uma coorte prospectiva de adultos, independentemente de sexo e etnia, que foram submetidos a CRM ou troca ou reconstrução valvar. Esses pacientes foram operados consecutivamente e eletivamente entre janeiro de 2016 e dezembro de 2017. Os critérios de exclusão foram: pacientes com mobilidade restrita secundária a condições ortopédicas ou neurológicas, portadores de angina instável, classificados na classe IV de acordo com a New York Heart Association Functional Classification (NYHA) no momento da operação e pacientes com diagnóstico de infarto agudo do miocárdio <30 dias no pré-operatório.

Realizada esternotomia mediana e circulação extracorpórea (CEC) em todos os pacientes. O circuito da CEC foi preparado com solução de Ringer e o fluxo da bomba foi ajustado para 2,4 L/min/m^2^. Nenhum paciente recebeu corticosteroides. Inotrópicos, vasopressores, nitratos e nitroprussiato de sódio foram administrados no intra ou pós-operatório, a critério do anestesiologista ou da equipe da unidade de terapia intensiva.

Este estudo foi aprovado pelo Comitê de Ética em Pesquisa do Hospital das Clínicas da Faculdade de Medicina de Ribeirão Preto-USP (registro 15363/2014) e o consentimento informado foi obtido de cada paciente incluído no estudo.

#### Avaliação de Fragilidade

A fragilidade foi avaliada por meio do índice de fragilidade de Fried, que inclui 5 critérios (teste de velocidade de marcha de 5 m, força de preensão manual, perda de peso, exaustão e inatividade). Aqueles que preencheram 3 desses 5 critérios foram diagnosticados com fragilidade. Os pacientes que atenderam a 2 dos 5 critérios foram considerados no estágio pré-frágil (um subconjunto com alto risco de progredir para fragilidade).^7^ A atividade física foi avaliada por meio do Questionário Internacional de Atividade Física.^8^

Para o teste de velocidade de marcha de 5 m, o paciente foi posicionado atrás da linha de partida de 5 m e instruído a caminhar em um ritmo confortável até alguns passos além da marca de 5 m. O cronômetro foi iniciado com a primeira passada após a linha de 0 m e interrompido com a primeira passada após a linha de 5 m. A força de preensão manual foi avaliada medindo-se o grau de força isométrica desenvolvido por meio de um dinamômetro manual mecânico (MN 70142-North Coast®).

A sarcopenia é conhecida por afetar a força dos músculos respiratórios; assim, avaliamos a pressão inspiratória e expiratória máxima por meio de um manovacuômetro digital portátil (Mvd 300®, Globalmed, Porto Alegre, Brasil). As medições foram realizadas com no mínimo 3 tentativas com um intervalo de 1 minuto entre cada repetição usando um clipe nasal. Qualquer valor que apresentasse variação >10% era desconsiderado e foram utilizadas as medidas com o valor mais alto.

#### Avaliação de Capacidade Funcional

O teste de caminhada de 6 minutos foi realizado com base nas recomendações da American Thoracic Society, declaração de 2002, para avaliar a capacidade funcional dos pacientes.^9^

#### Desfechos

Óbito por qualquer causa até 1 mês após a alta hospitalar foi considerado mortalidade hospitalar pós-operatória, que foi o desfecho primário deste estudo. Nossos desfechos secundários foram a incidência de infecção(ões) pós-operatória e disfunção respiratória, renal ou cardiovascular.

#### Análise Estatística

A distribuição dos dados foi verificada pela análise dos histogramas de distribuição, gráficos Q-Q e teste de Shapiro-Wilk. Como nossos dados tinham distribuições não normais, optamos por usar métodos estatísticos não paramétricos. Os resultados foram apresentados como mediana e intervalo interquartil para variáveis contínuas e percentuais para variáveis categóricas. Para as comparações de variáveis categóricas, utilizou-se o teste exato de Fisher. O teste de Mann-Whitney ou Kruskal-Wallis seguido do teste post hoc de Dunn-Bonferroni foram usados para comparar os dados contínuos. O valor de p<0,05 foi considerado estatisticamente significativo. A análise estatística foi feita no programa Statistical Package for the Social Sciences (IBM-SPSS) SPSS 17 versão 22.0.

## Resultados

Foram incluídos 100 pacientes: 59 foram submetidos à cirurgia valvar cardíaca e 41 à CRM. A Tabela 1 mostra as características clínicas dos pacientes.

**Tabela 1:** Características clínicas de acordo com a valvopatia

	Valvopatia n=59	DAC n=41	p
Idade (anos)	54 (45–61)	62 (55–69)	0,002
Mulheres	60%	29%	0,013
Peso	75 (64–87)	79 (72–86)	0,104
Altura (cm)	164 (154–173)	165 (158–170)	0,501
Índice de massa corporal	29 (24–32)	29 (25–32)	0,342
Hipertensão arterial	55,0%	82,4%	0,062
Diabetes melito	15,0%	64,7%	<0,001
Acidente vascular cerebral	8%	2%	0,211
Histórico de IM	34%	4%	<0,001
Fibrilação atrial crônica	25,0%	0,0%	<0,001
Tabagismo	15,0%	35,3%	0,272
Hipertensão pulmonar	17,5%	0,0%	0,005
Insuficiência renal	5,0%	0,0%	0,211
Cirurgia prévia	35,0%	5,9%	<0,001
Classe NYHA			
I	0,0%	5,9%	
II	62,5%	52,9%	0,230
III	37,5%	41,2%	
IV	0,0%	0,0%	
Fração de ejeção	60 (48–66)	51 (44–63)	0,028
Creatinina sérica (dg/l)	1,1 (0,9–1,4)	1,2 (1,1–1,3)	0,484
Hemoglobina	13 (12–14)	13 (12–14)	0,793

DAC: doença arterial coronariana; IM: infarto do miocárdio. Teste exato de Fisher (variáveis categóricas) e Mann-Whitney (dados contínuos).

O tempo de CEC para cirurgia valvar e CRM foi de 122 min (100–165 min) e 110 (77–130 min), respectivamente (p=0,002), e o tempo de pinçamento aórtico foi de 93 min (76–117 min) e 72 (49–95 min), respectivamente (p<0,001).

A idade mediana de toda a coorte foi de 57 anos (49–66 anos) e 57% dos pacientes tinham menos de 60 anos. No geral, com base nos critérios de fragilidade de Fried, 17 pacientes (17%) foram considerados frágeis, 70 (70%) foram considerados pré-frágeis e apenas 13 (13%) foram considerados não frágeis.

Entre os portadores de valvopatia, 11 (18,6%) foram considerados frágeis e 43 (73%) foram considerados pré-frágeis. O percentual de pacientes com fragilidade e pré-fragilidade entre os que realizaram CRM foi de 6 (14,6%) e 27 (66%), respectivamente. A diferença no percentual de fragilidade entre os pacientes com doença valvar e coronariana não foi significativa (p=0,788).

O tempo de CEC foi 110 min (85–135 min), 120 (95–147 min) e 107 min (75–145 min), respectivamente, em pacientes não frágeis, pré-frágeis e frágeis (p=0,656), enquanto o tempo de pinçamento aórtico foi de 76 min (67–100 min), 90 (71–113 min) e 86 min (46–114 min), respectivamente (p=0,361).

A distribuição dos critérios de Fried entre pacientes com valvopatia e doença arterial coronariana (DAC) é mostrada na Tabela 2. O grupo com doença valvar cardíaca apresentou proporção significativamente maior de pacientes com alteração do peso corporal e o tempo de transição (5 min) foi significativamente maior no grupo DAC.

**Tabela 2:** Distribuição dos critérios de Fried de acordo com a doença cardíaca

	Todos (%)	Valvopatia n=59	DAC n=41	p (DVC x DAC)
Alteração do peso corporal	14%	20%	5%	0,028
Exaustão	63%	68%	56%	0,223
Atividade física	62%	68%	54%	0,152
Tempo de transição (seg.)	4,7 (4,0–5,7)	4,5 (4,0–5,3)	5,1 (4,4–6,0)	0,019
Força de preensão manual (kgf)	28 (20–35)	28 (20–35)	29 (20–36)	0,615

DCV: doença valvar cardíaca; Teste exato de Fisher (variáveis categóricas) e Mann-Whitney (dados contínuos)

A Tabela 3 mostra as características antropométricas, distribuição das comorbidades associadas, critérios de fragilidade de Fried, pressões respiratórias e estado funcional (classe NYHA, fração de ejeção do ventrículo esquerdo, distância do TC6M) entre os pacientes não frágeis, pré-frágeis e frágeis, independentemente da doença cardíaca. Entre os pacientes frágeis, 53% tinham menos de 60 anos (mediana=48 anos, 44–54 anos). A proporção de pacientes com infarto do miocárdio prévio entre pacientes não frágeis, pré-frágeis e frágeis com DAC foi de 50%, 37% e 50%, respectivamente. As etiologias da valvopatia foram febre reumática em 44%, degenerativa em 37%, endocardite em 5% e dilatação anular por aumento ventricular em 13%. A Tabela 4 mostra a etiologia e o mecanismo da disfunção valvar de acordo com o fenótipo de Fried.

**Tabela 3:** Características clínicas de acordo com o fenótipo de Fried

	Não frágil n=13	Pré-frágil n=70	Frágil n=17	p
Idade	58 (51–60)	57 (49–66)	57 (48–69)	0,830
Mulheres	38%	44%	76%	0,042
Índice de massa corporal	28 (25–30)	29 (25–32)	30 (24–34)	0,617
Hipertensão arterial	92%	70%	71%	0,243
Diabetes melito	37%	30%	35%	0,793
Histórico de IM	31%	16%	23%	0,389
Acidente vascular cerebral prévio	8%	7%	0%	0,519
Insuficiência renal	0%	8%	0%	0,255
HSAP	8%	11%	6%	0,757
Fibrilação atrial	8%	18%	17%	0,629
Cirurgia cardíaca prévia	8%	20%	29%	0,338
Classe NYHA				
I	8%	0%	6%	
II	61%	67%	47%	0,163
III	31%	33%	47%	
IV	0%	0%	0%	
Fração de ejeção	56 (46–60)	58 (47–65)	60 (45–68)	0,605
Creatinina sérica (dg/l)	1,1 (1,0–1,1)	1,2 (0,9–1,4)	1,1 (1,0–1,2)	0,565
Distância do TC6M (m)	417 (384–482)	423 (360–493)	307 (275–364)	0,005**
PIM (cm H_2_O)	65 (52–106)	70 (49–96)	44 (40–66)	0,015*
PEM (cm H_2_O)	77 (58–123)	90 (73–118)	64 (56–108)	0,160
Alteração do peso corporal	0%	9%	47%	<0,001**
Exaustão	0%	66%	94%	<0,001*
Baixa atividade física	0%	69%	88%	<0,001*
Teste de velocidade de marcha de 5 m (seg.)	4,6 (3,5–4,7)	4,7 (4,0–5,6)	6,7 (5,3–8,4)	<0,001**
Força de preensão manual (kgf)	34 (23–45)	30 (21–37)	20 (15–27)	0,018**

* significativo para frágeis vs. não frágeis;

** significativo para frágeis vs. não frágeis e pré-frágeis. Teste exato de Fisher (variáveis categóricas) e teste de Kruskal-Wallis seguido de teste post hoc de Dunn-Bonferroni (dados contínuos). IM: infarto do miocárdio; HSAP: Hipertensão sistólica arterial pulmonar. Teste exato de Fisher (variáveis categóricas). PIM: pressões inspiratórias máximas; PEM: pressões expiratórias máximas.

**Tabela 4:** Etiologia e disfunção das doenças valvares

		FRIED					
Disfunção valvar		Não frágil		Pré-frágil		Frágil	
		n	%	n	%	n	%
	Estenose mitral	0	0,0%	1	2,3%	0	0,0%
	Regurgitação mitral	1	20,0%	8	18,6%	3	27,2%
	Insuficiência aórtica	1	20,0%	1	2.3%	0	0,0%
	Estenose aórtica	0	0,0%	3	7,0%	4	36,4%
	Dupla lesão mitral	0	0,0%	5	11,6%	0	0,0%
	Dupla lesão aórtica	1	20,0%	4	9,3%	0	0,0%
	Disfunção mitroaórtica	2	40,0%	21	48,8%	4	36,4%
							
	Degenerativa	2	40,0%	13	30,2%	5	45.5%
Etiologia	Reumática	1	20,0%	19	44,2%	3	27,3%
	Endocardite	0	0,0%	2	4,7%	1	9,1%
	RM secundária	1	20,0%	6	14,0%	1	9,1%
	Outros						

A Tabela 5 mostra as características da valvopatia de acordo com o fenótipo de Fried.

**Tabela 5:** Evolução pós-operatória de acordo com a escala de Fried

	Não-frágil n=13	Pré-frágil n=70		p
Óbito hospitalar	0%	8,6%	29,4%	0,026
Tempo de terapia intensiva (dias)	3 (2–3)	3 (2–5)	3 (2–4)	0,946
Tempo de hospitalização (dias)	10 (7–11)	11 (7–16)	9 (6–22)	0,861
Tempo de VMI (hora)	15 (5–19)	17 (7–28)	13 (7–12)	0,615
Creatinina	1,2 (0,9–1,2)	1,3 (1,0–1,7)	1,1 (1,0–1,4)	0,231
Hemoglobina	10 (9–11)	10 (9–11)	10 (10–11)	0,994
Ventilação não invasiva	38%	40%	23%	0,448
Congestão pulmonar cardiogênica	46%	41%	47%	0,888
Pneumonia	8%	8%	23%	0,193
Infecção do trato urinário	0,0%	13%	23%	0,164
Infecção superficial da lesão	8%	6%	12%	0,667
Mediastinite	0%	3%	6%	0,640
Disfunção renal aguda	0,0%	8%	0%	0,255
Acidente vascular cerebral	0,0%	3%	12%	0,178

Teste exato de Fisher (variáveis categóricas). VMI: Ventilação mecânica invasiva.

A mortalidade hospitalar geral foi de 11%, 12% para valvopatia e 10% para CRM (p=0,762). A mortalidade hospitalar foi significativamente maior em pacientes frágeis (29,4%, p=0,026) do que em pacientes pré-frágeis (8,6%) e não frágeis (0%). Não foram observadas diferenças estatisticamente significativas na porcentagem de complicações intra-hospitalares (Tabela 5).

## Discussão

Nossos resultados mostraram que, no geral, 17% dos pacientes estudados eram frágeis e 70% pré-frágeis, apesar do predomínio de não idosos (<60 anos) na coorte estudada (57%). Além disso, exceto pela maior proporção de mulheres entre os pacientes frágeis, os pacientes frágeis, pré-frágeis e não frágeis foram semelhantes quanto à distribuição das comorbidades, função ventricular esquerda e classificação NYHA.

Sabe-se que a fragilidade está associada a resultados pós-operatórios adversos em pacientes submetidos à cirurgia cardíaca.^10^^–^^14^ No entanto, a maioria dos estudos relatou fragilidade em pacientes idosos.^6^^,^^10^^,^^13^^,^^14^ Nossos achados demonstram que a fragilidade ocorre em uma porcentagem significativa de pacientes não idosos com diagnóstico de valvopatia ou coronariopatia submetidos à cirurgia cardíaca a céu aberto. Esse fato pode ser atribuído à associação de fatores socioeconômicos com doenças crônicas, representando o conceito de “fragilidade secundária”, termo utilizado para se referir à fragilidade na presença de doenças crônicas.^15^ Os pacientes frágeis observados em nosso estudo não diferiram dos demais quanto à presença de comorbidades e estado funcional cardíaco. Diferenciaram-se essencialmente nos aspectos psicológicos e também no que diz respeito à sarcopenia, evidenciada pelas alterações relatadas do peso corporal e pela baixa atividade física e mobilidade.

Embora geralmente vista como uma característica da velhice, a fragilidade tem sido descrita em pessoas não idosas, principalmente entre os estratos socioeconômicos mais baixos das sociedades.^16^^,^^17^ Santos-Eggimann et al.,^17^ analisaram 18.227 comunidades europeias selecionadas aleatoriamente e observaram 4,1% de frágeis e 37,4% de pré-frágeis em uma população de meia-idade. Além disso, observaram uma forte relação entre escolaridade e fragilidade. Brothers et al.,^18^ observaram níveis mais elevados de fragilidade entre imigrantes europeus de meia-idade e mais velhos nascidos em países de baixa e média renda, sugerindo também que fatores socioeconômicos podem influenciar significativamente a saúde de um indivíduo ao longo de sua vida.

A avaliação de risco nesses pacientes tem sido uma preocupação entre os cirurgiões cardiotorácicos. Consequentemente, diferentes sistemas de pontuação de risco foram desenvolvidos para prever mortalidade e morbidade.^19^^–^^21^ No entanto, a maioria dos sistemas de pontuação enfatiza disfunções orgânicas específicas e aspectos operacionais, com menor ênfase nas consequências físicas e psicológicas das doenças crônicas e nos efeitos da dimensão socioeconômica. Além disso, especulamos que a alta mortalidade encontrada no grupo dos frágeis (29,4%), comparada a informações encontradas na literatura, pode ser decorrente da associação entre fragilidade e condições crônicas preexistentes.^22^^–^^24^

Nossos achados demonstram que, além das comorbidades e disfunções de órgãos específicos, o fenótipo da fragilidade deve ser considerado um fator importante para avaliação do risco operatório, pois pode refletir não apenas as consequências de uma doença crônica, mas também a dimensão socioeconômica. Portanto, o fenótipo da fragilidade, mesmo em não idosos, pode contribuir para oferecer uma visão mais holística do estado de saúde do paciente que possa auxiliar no desenvolvimento de ações de intervenção da equipe multiprofissional.

Diante desses fatos, é importante, no tratamento de pacientes frágeis (idosos e não idosos), decidir se eles se beneficiariam do adiamento da operação para iniciar um programa de reabilitação multidisciplinar pré-operatório. Pesquisas baseadas em evidências mostram que vários aspectos relacionados à fragilidade, como sarcopenia, sedentarismo e problemas nutricionais, são potencialmente tratáveis^25^^–^^27^ e podem diminuir a mortalidade operatória.^28^^,^^29^

Waite et al.^29^ demonstraram conclusivamente que um programa de reabilitação pré-operatória domiciliar para pacientes frágeis com idade ≥65 anos submetidos a CRM ou cirurgia valvar pode melhorar seu estado funcional e reduzir o tempo de hospitalização. Além disso, as evidências sugerem que a preparação psicológica pode ajudar a reduzir a dor pós-operatória, os efeitos negativos e o tempo de hospitalização, bem como melhorar a recuperação comportamental,^16^ e, além de exercícios, um programa de suporte nutricional pré-operatório pode possivelmente reduzir a sarcopenia e melhorar os resultados pós-operatórios.^27^^,^^30^

Embora pesquisas baseadas em evidências sugiram a superioridade de programas de exercícios multicomponentes sobre um programa de exercícios de um único componente para a reabilitação de pacientes frágeis,^31^ um programa mais focado voltado para disfunções orgânicas específicas pode ser benéfico. Katsura et al.,^32^ relataram que o treinamento muscular inspiratório pré-operatório esteve associado à redução da atelectasia pós-operatória, pneumonia e tempo de hospitalização em adultos submetidos a cirurgia cardíaca e abdominal de grande porte. No entanto, apesar dos efeitos favoráveis que um programa de exercícios pode proporcionar, é necessário considerar os riscos e incertezas associados à frequência, tipo e duração do exercício para pacientes frágeis com diagnóstico de doença cardíaca para os quais uma operação cardíaca é essencial.

Acreditamos que nosso estudo seja um dos primeiros a investigar a prevalência de fragilidade em pacientes não idosos com diagnóstico de valvopatia ou coronariopatia submetidos à cirurgia cardíaca e suas consequências na evolução hospitalar.

As limitações de nosso estudo são a coorte de pequeno porte, que pode não fornecer o poder estatístico necessário para comentar sobre a significância dos achados/resultados ou para analisar os fatores de confusão. Além disso, uma pequena coorte pode não incluir todas as complicações pós-operatórias que podem estar associadas à fragilidade. Portanto, estudos futuros são necessários para corroborar nossos achados e verificar a relação risco-benefício do uso de programas de reabilitação pré-operatória para pacientes frágeis.

## Conclusão

Observamos que a fragilidade é prevalente mesmo em pacientes não idosos submetidos a CRM ou cirurgia valvar e está associada a maior mortalidade hospitalar pós-operatória.
