# Supplementary material for: Fragilidade entre Pacientes não Idosos Submetidos à Cirurgia Cardíaca
Source: Arq Bras Cardiol. 2020 Oct 13;115(4):604–10. [Article in Portuguese] doi: 10.36660/abc.20190082 (PMC8386982; doi:10.36660/abc.20190082)
Supplement: Translation

# Translation: Frailty Among Non-Elderly Patients Undergoing Cardiac Surgery

**Authors:** Camila Bottura, Livia Arcêncio, Hannah Miranda Araújo Chagas, Paulo Roberto Barbosa Evora, Alfredo José Rodrigues

**Affiliations:** 1 São PauloSP Brazil Faculdade de Medicina de Ribeirão Preto - Universidade de São Paulo, Ribeirão Preto, São Paulo, SP - Brazil

**Keywords:** Frailty, Myocardial Revascularization/surgery, Heart Valves/surgery, Postoperative Care/mortality

## Abstract

**Background:**

Usually viewed as a characteristic of old age, frailty may also occur in non-elderly people, primarily in those suffering from chronic disease. Frailty may increase operative risk.

**Objectives:**

To determine the prevalence of frailty patients undergoing coronary artery bypass (CABG) and/or heart valve replacement or reconstruction and/or heart valve surgery, as well as the influence of frailty on postoperative outcomes.

**Methods:**

Our study comprised 100 adults who underwent consecutive elective cardiac operations. Frailty was assessed using the Fried scale. Patients also performed a 6-minute walk test, and we measured maximal inspiratory and expiratory pressures. A p value <0.05 was considered significant.

**Results:**

Of a cohort of 100 patients, based on the Fried frailty criteria, 17 patients (17%) were considered frail, 70 (70%) pre-frail and only 13 (13%) were non-frail. Among patients with valvular heart disease, 11 (18.6%) were considered frail and 43 (73%) pre-frail. Fifty three percent of the patients considered frail were less than 60 years old (median=48 years old). The differences in frailty phenotype between patients with valvular heart disease and coronary artery disease were not statistically significant (p=0.305). A comparison between non-frail, pre-frail, and frail patients showed no significant difference in the distribution of comorbidities and cardiac functional status, regardless of their cardiac disease. However, hospital mortality was significantly higher in frail patients (29.4%, p=0.026) than in pre-frail patients (8.6%) and non-frail patients (0%).

**Conclusions:**

Frailty is prevalent even among non-elderly patients undergoing CABG or valvular heart surgery and is associated with higher postoperative hospital mortality.

## Introduction

Frailty is a syndrome of increased vulnerability to stressors including hospitalization and is associated with a reduced physiological reserve secondary to a decline in the optimal function of multiple physiological systems, which predisposes individuals to high risk of adverse events.^1^ It is a multidimensional syndrome comprising physical, psychological and social dimensions,^2^ usually viewed primarily as a geriatric syndrome^3^ characterized by low physical activity, muscle weakness, slowed performance, fatigue or poor endurance and unintentional weight loss. In this context, the Fried criteria are widely used for the physical domains of frailty, which can be easily interpreted by non-geriatricians and may have prognostic value.^4^^,^ ^5^ Association of frailty with chronic comorbidities has been shown.^2^^,^^6^ Therefore, even non-elderly patients may present with this condition.

We have observed, in the course of our clinical practice, that several non-elderly patients undergoing open heart surgery demonstrate clinical features compatible with frailty, usually experiencing less favorable postoperative hospital outcomes.

Therefore, the objective of the present study was to determine the prevalence of frailty in non-elderly patients undergoing coronary artery bypass (CABG) and/or heart valve surgery, as well as to assess the influence of frailty on postoperative hospital outcomes.

### Patients and Methods

#### Study Design and Participants

We studied a prospective cohort of adults regardless of gender and race, who underwent CABG or heart valve replacement or reconstruction. These patients were consecutively and electively operated between January 2016 and December 2017. Exclusion criteria were: patients with restricted mobility secondary to orthopedic or neurological conditions, those with unstable angina, those classified as class IV as per the New York Heart Association Functional Classification (NYHA) at the time of the operation, and patients diagnosed with acute myocardial infarction <30 days preoperatively.

Median sternotomy and cardiopulmonary bypass (CPB) were performed on all patients. The CPB circuit was primed using Ringer's solution, and the pump flow was adjusted to 2.4 L/min/m^2^. No patient received corticosteroids. Inotropes, vasopressors, nitrates and sodium nitroprusside were administered intra- or postoperatively at the discretion of the anesthesiologist or the intensive care unit staff.

This study was approved by the institutional review board of Hospital das Clínicas da Faculdade de Medicina de Ribeirão Preto-USP (registry 15363/2014) and informed consent was obtained from each patient included in the study.

#### Assessment of Frailty

Frailty was assessed using the Fried frailty index, which includes 5 criteria (5-m gait speed test, handgrip strength, weight loss, exhaustion and inactivity). Those who met 3 of these 5 criteria were diagnosed with frailty. Patients who met 2 of the 5 criteria were considered as being in the pre-frail stage (a subset at high risk of progressing to frailty).^7^ Physical activity was assessed using the International Physical Activity Questionnaire.^8^

For the 5-m gait speed test, the patient was positioned behind the 5-m start line and instructed to walk at a comfortable pace until a few steps past the 5-m mark. The timer was started with the first footfall after the 0-m line and stopped with the first footfall after the 5-m line. Handgrip strength was assessed by measuring the degree of isometric strength developed using a mechanical hand dynamometer (MN 70142-North Coast®).

Sarcopenia is known to affect respiratory muscle strength; thus, we evaluated maximal inspiratory and expiratory pressure using a portable digital manovacuometer (Mvd 300®, Globalmed, Porto Alegre, Brazil). Measurements were performed over a minimum of 3 trials with a 1-min interval between each repetition using a nasal clip. Any value showing >10% variation was disregarded, and measurements with the highest value were used.

#### Functional Capacity Assessment

A 6-minute walk test was performed based on the recommendations of the American Thoracic Society, 2002 statement, to assess the functional capacity of patients.^9^

#### Outcomes

Death from any cause until 1 month after hospital discharge was considered postoperative hospital mortality, which was the primary outcome measure of this study. Our secondary outcomes were the incidence of postoperative infection(s), and respiratory, renal or cardiovascular dysfunction.

#### Statistical Analysis

Data distribution was verified by analysis of distribution histograms, Q-Q graphs and the Shapiro-Wilk test. Since our data had non-normal distributions, we chose to use non-parametric statistical methods. Results were presented as median and interquartile range for continuous variables and percentages for categorical variables. Fisher's Exact Test was used to compare categorical variables. Mann-Whitney or Kruskal-Wallis test followed by a Dunn-Bonferroni post hoc test were used to compare continuous data. A p value <0.05 was considered statistically significant. Statistical analysis was performed using the software Statistical Package for the Social Sciences (IBM-SPSS) version 22.0.

## Results

Our study included 100 patients: 59 underwent heart valve surgery and 41 underwent CABG. Table 1 shows the clinical characteristics of patients.

**Table 1:** Clinical characteristics according to the heart disease

	VHD n=59	CAD n=41	p
Age (y)	54 (45–61)	62 (55–69)	0.002
Female	60%	29%	0.013
Weight	75 (64–87)	79 (72–86)	0.104
Height (cm)	164 (154–173)	165 (158–170)	0.501
Body mass index	29 (24–32)	29 (25–32)	0.342
Arterial hypertension	55.0%	82.4%	0.062
Diabetes mellitus	15.0%	64.7%	<0,001
Stroke	8%	2%	0.211
Previous MI	34%	4%	<0.001
Chronic atrial fibrillation	25.0%	0.0%	<0.001
Smoke	15.0%	35.3%	0.272
Pulmonary hypertension	17.5%	0.0%	0.005
Kidney failure	5.0%	0.0%	0.211
Previous operation	35.0%	5.9%	<0.001
NYHA class			
I	0.0%	5.9%	
II	62.5%	52.9%	0.230
III	37.5%	41.2%	
IV	0.0%	0.0%	
Ejection fraction	60 (48–66)	51 (44–63)	0.028
Serum creatinine (dg/l)	1.1 (0.9–1.4)	1.2 (1.1–1.3)	0.484
Hemoglobin	13 (12–14)	13 (12–14)	0.793

CAD: coronary artery disease; VHD: valvular heart disease; MI: myocardial infarction. Fisher's Exact Test (categorical variables) and Mann-Whitney (continuous data)

CPB time for valvular surgery and CABG was 122 min (100–165 min) and 110 (77–130 min), respectively (p=0.002), and the aortic cross-clamp time was 93 min (76–117 min) and 72 (49–95 min), respectively (p<0.001).

The median age for the whole cohort was 57 years (49-66 years) and 57% of the patients were younger than 60. Overall, based on the Fried frailty criteria, 17 patients (17%) were considered frail, 70 (70%) were considered pre-frail and only 13 (13%) were non-frail.

Among patients with valvular heart disease, 11 (18.6%) were considered frail and 43 (73%) were considered pre-frail. The percentage of patients showing frailty and pre-frailty among those who underwent CABG was 6 (14.6%) and 27 (66%), respectively. The difference in the percentage of frailty between patients with valvular and coronary artery disease was not significant (p=0.788).

CPB time was 110 min (85–135 min), 120 (95–147 min) and 107 min (75–145 min), respectively, in non-frail, pre-frail and frail patients (p=0.656), whereas aortic cross-clamp time was 76 min (67–100 min), 90 (71–113 min) and 86 min (46–114 min), respectively (p=0.361).

Distribution of Fried criteria between patients with valvular heart disease (VHD) and coronary artery disease (CAD) is shown in Table 2. The heart valvular disease group had significantly higher proportion of patients with change of body weight and transition time (5 min) was significantly longer in the CAD group.

**Table 2:** Fried's criteria distribution to according the heart disease

	All (%)	VHD n=59	CAD n=41	p (VHDxCAD)
Change in body weight	14%	20%	5%	0.028
Exhaustion	63%	68%	56%	0.223
Low physical activity	62%	68%	54%	0.152
Transition time (sec.)	4.7 (4.0–5.7)	4.5 (4.0–5.3)	5.1 (4.4–6.0)	0.019
Handgrip strength (kgf)	28 (20–35)	28 (20–35)	29 (20–36)	0.615

CAD: coronary artery disease; VHD: valvular heart disease; HVD: heart valve disease. Fisher's exact Test (categorical variables) and Mann-Whitney (continuous data)

Table 3 shows anthropometric characteristics, distribution of associated comorbidities, Fried frailty criteria, respiratory pressures and functional status (NYHA class, left ventricular ejection fraction, 6MWT distance) among non-frail, pre-frail and frail patients, regardless of the cardiac disease. Among frail patients, 53% were less than 60 years old (median=48 years, 44–54 years). The proportion of patients who experienced previous myocardial infarction among non-frail, pre-frail and frail patients with CAD was 50%, 37% and 50%, respectively. Etiologies of valve disease were rheumatic fever in 44%, degenerative in 37%, endocarditis in 5% and annular dilation due to ventricular enlargement in 13%. Table 4 shows the etiology and mechanism of valve dysfunction according the Fried phenotype.

**Table 3:** Clinical characteristics to according the Fried's phenotype

	Non-Frail n=13	Pre-frail n=70	Frail n=17	p
Age	58 (51–60)	57 (49–66)	57 (48–69)	0.830
Female	38%	44%	76%	0.042
Body mass index	28 (25–30)	29 (25–32)	30 (24–34)	0.617
Arterial hypertension	92%	70%	71%	0.243
Diabetes mellitus	37%	30%	35%	0.793
Previous MI	31%	16%	23%	0.389
Previous stroke	8%	7%	0%	0.519
Kidney failure	0%	8%	0%	0.255
SAPH	8%	11%	6%	0.757
Atrial fibrillation	8%	18%	17%	0.629
Previous cardiac operation	8%	20%	29%	0.338
NYHA class				
I	8%	0%	6%	
II	61%	67%	47%	0.163
III	31%	33%	47%	
IV	0%	0%	0%	
Ejection fraction	56 (46–60)	58 (47–65)	60 (45–68)	0.605
Serum creatinine (dg/l)	1.1 (1.0–1.1)	1.2 (0.9–1.4)	1.1 (1.0–1.2)	0.565
6MWT distance (m)	417 (384–482)	423 (360–493)	307 (275–364)	0.005**
MIP (cm H_2_O)	65 (52–106)	70 (49–96)	44 (40–66)	0.015*
MEP (cm H_2_O)	77 (58–123)	90 (73–118)	64 (56–108)	0.160
Change in body weight	0%	9%	47%	<0.001**
Exhaustion	0%	66%	94%	<0.001*
Low physical activity	0%	69%	88%	<0.001*
5-m gait speed test (sec)	4.6 (3.5–4.7)	4.7 (4.0–5.6)	6.7 (5.3–8.4)	<0.001**
Handgrip strength (kgf)	34 (23–45)	30 (21–37)	20 (15–27)	0.018**

* significant for frail vs. non-frail;

** significant for frail vs. non-frail and pre-frail. Fisher's exact test (categorical variables) and Kruskal-Wallis test followed by a Dunn-Bonferroni post hoc test (continuous data). IQR: interquartile range. Fisher's exact test (categorical variables). MI: myocardial infarction; SAPH: systolic arterial pulmonary hypertension; MIP: maximal inspiratory pressures; MEP: maximal expiratory pressures.

**Table 4:** Etiology and dysfunction of valve diseases

		FRIED					
Valve dysfunction		Non-frail		Pre-frail		Frail	
		n	%	n	%	n	%
	Mitral stenosis	0	0.0%	1	2.3%	0	0.0%
	Mitral regurgitation	1	20.0%	8	18.6%	3	27.2%
	Aortic regurgitation	1	20.0%	1	2.3%	0	0.0%
	Aortic stenosis	0	0.0%	3	7.0%	4	36.4%
	Double mitral lesion	0	0.0%	5	11.6%	0	0.0%
Etiology	Double aortic lesion	1	20.0%	4	9.3%	0	0.0%
	Mitro-aortic dysfunction	2	40.0%	21	48.8%	4	36.4%
	Degenerative	2	40.0%	13	30.2%	5	45.5%
	Rheumatic	1	20.0%	19	44.2%	3	27.3%
	Endocarditis	0	0.0%	2	4.7%	1	9.1%
	Secondary MR	1	20.0%	6	14.0%	1	9.1%
	Other						

Table 5 shows the valve disease characteristics according to the Fried phenotype.

**Table 5:** Postoperative evolution according to the Fried's scale

	Non-frail n=13	Pre-frail n=70	Frail n=17	p
Hospital death	0%	8.6%	29.4%	0.026
Intensive care time (days)	3 (2–3)	3 (2–5)	3 (2–4)	0.946
Hospital time (days)	10 (7–11)	11 (7–16)	9 (6–22)	0.861
IMV time (hour)	15 (5–19)	17 (7–28)	13 (7–12)	0.615
Creatinine	1.2 (0.9–1.2)	1.3 (1.0–1.7)	1.1 (1.0–1.4)	0.231
Hemoglobin	10 (9–11)	10 (9–11)	10 (10–11)	0.994
Non-invasive ventilation	38%	40%	23%	0.448
Cardiogenic pulmonary congestion	46%	41%	47%	0.888
Pneumonia	8%	8%	23%	0.193
Urinary infection	0.0%	13%	23%	0.164
Superficial wound infection	8%	6%	12%	0.667
Mediastinitis	0%	3%	6%	0.640
Acute kidney failure	0.0%	8%	0%	0.255
Stroke	0.0%	3%	12%	0.178

IQR: interquartile range. Fisher's exact test (categorical variables). IMV: Invasive mechanical ventilation.

Overall hospital mortality was 11%, 12% for valvular heart disease and 10% for CABG (p=0.762). Hospital mortality was significantly higher in frail patients (29.4%, p=0.026) than in pre-frail patients (8.6%) and non-frail patients (0%). No statistically significant differences were observed in the percentage of in-hospital complications (Table 5).

## Discussion

Our results have shown that, overall, 17% of patients studied were frail and 70% pre-frail, despite the fact that non-elderly (<60 years) prevailed in the cohort studied (57%). In addition, except for the higher proportion of women among frail patients, frail, pre-frail and non-frail patients were similar regarding the distribution of comorbidities, left ventricle function and NYHA class.

It is well known that frailty is associated with adverse postoperative outcomes in those undergoing cardiac surgery.^10^^–^^14^ However, most studies have reported frailty in elderly patients.^6^^,^^10^^,^^13^^,^^14^ Our findings demonstrate that frailty occurs in a significant percentage of non-elderly patients diagnosed with valvular heart disease or coronary artery disease undergoing open heart surgery. This fact can be attributed to the association of socioeconomic factors with chronic disease, representing the concept of “secondary frailty,” a term used to refer to frailty in the presence of chronic diseases.^15^ The frail patients observed in our study did not differ from others in terms of the presence of comorbidities and cardiac functional status. They differed essentially in terms of psychological aspects and also in terms of sarcopenia, evidenced by the reported changes in body weight and low physical activity and mobility.

Although usually viewed as a characteristic of old age, frailty has been described in non-elderly people, primarily among the lower socioeconomic strata in societies.^16^^,^^17^ Santos-Eggimann et al.^17^ analyzed 18,227 randomly selected European communities and observed 4.1% frail and 37.4% pre-frail individuals in a middle-aged population. Moreover, they observed a strong relationship between education and frailty. Brothers et al.^18^ observed higher levels of frailty among middle-aged and older European immigrants born in low- and middle-income countries, also suggesting that socioeconomic factors may significantly influence an individual's health throughout his/her life.

Risk assessment in these patients has been a concern among cardiothoracic surgeons. Consequently, different risk scoring systems have been developed to predict mortality and morbidity.^19^^–^^21^ However, most scoring systems emphasize specific organic dysfunction and aspects of operation, with lesser emphasis on the physical and psychological consequences of chronic diseases and the effects of the socioeconomic dimension. In addition, we speculate that the high mortality found in the fragile group (29.4%), compared to previous information found in the literature, may result from the association between fragility and the preexisting chronic conditions.^22^^–^^24^

Our findings demonstrate that in addition to comorbidities and specific organ dysfunctions, frailty phenotype should be considered an important factor for operative risk assessment, because it may reflect not only the consequences of a chronic disease, but also the socioeconomic dimension. Therefore, frailty phenotype, even in non-elderly, may contribute toward offering a more holistic view of the patient's health status that can assist the development of intervention actions of the multi-professional team.

In view of these facts, it is important when treating frail patients (both elderly and non-elderly) to decide if they would benefit from postponing their operation to engage in a preoperative multidisciplinary rehabilitation program. Evidence-based research has shown that several aspects related to frailty such as sarcopenia, physical inactivity and nutritional issues are potentially treatable^25^^–^^27^ and might decrease operative mortality.^28^^,^^29^

Waite et al.^29^ conclusively demonstrated that a home-based preoperative rehabilitation program for frail patients aged ≥65 years undergoing CABG or valvular surgery may improve their functional status and reduce the duration of hospitalization. Additionally, evidence suggests that psychological preparation may help reduce postoperative pain, negative effects, and the length of hospitalization, as well as improve behavioral recovery,^16^ and, besides exercise, a preoperative nutritional support program can potentially reduce sarcopenia and improve postoperative outcomes.^27^^,^^30^

Although evidence-based research suggests the superiority of multicomponent exercise programs over a single component exercise program for the rehabilitation of frail patients,^31^ a more focused program aimed at specific organ dysfunction may prove beneficial. Katsura et al.^32^ reported that preoperative inspiratory muscle training was associated with reduced postoperative atelectasis, pneumonia and length of hospitalization in adults undergoing cardiac and major abdominal surgery. However, notwithstanding the favorable effects that an exercise program may provide, it is necessary to consider the risks and uncertainty associated with frequency, type, and duration of exercise for frail patients diagnosed with heart disease in whom a cardiac operation is essential.

We believe that our study is one of the first to investigate the prevalence of frailty among non-elderly patients diagnosed with valvular heart disease or coronary artery disease undergoing cardiac surgery and its consequences with regard to hospital outcomes.

The limitations of our study are the small-sized cohort, which might not provide the required statistical power to comment on the significance of findings/results or to analyze confounders. Moreover, a small cohort may not include all postoperative complications that may be associated with frailty. Therefore, future studies are necessary to corroborate our findings and to verify the risk-benefit ratio of using preoperative rehabilitation programs for frail patients.

## Conclusion

We observed that frailty is prevalent even among non-elderly patients undergoing CABG or valvular heart surgery and is associated with higher postoperative hospital mortality.
